# Skeletal Characteristics and Clinical Treatment Patterns in Orthognathic Surgery: A Virtual Surgical Planning-Based Study

**DOI:** 10.3390/healthcare14060809

**Published:** 2026-03-22

**Authors:** Merve Berika Kadıoğlu, Mehmet Emre Yurttutan, Mehmet Alp Eriş, Meyra Durmaz, Ömer Faruk Kocamaz

**Affiliations:** 1 Department of Orthodontics, Faculty of Dentistry, Ankara University, 06560 Ankara, Türkiye; mkadioglu@ankara.edu.tr (M.B.K.); dtmeyradurmaz@gmail.com (M.D.); 2 Department of Oral and Maxillofacial Surgery, Faculty of Dentistry, Ankara University, 06560 Ankara, Türkiye; mehmet.alp.eris@gmail.com (M.A.E.); omerf.kocamaz@gmail.com (Ö.F.K.)

**Keywords:** 3D technologies, computer-aided design, dentoskeletal deformities, orthognathic surgery, virtual surgical planning

## Abstract

**Background/Objectives:** Virtual surgical planning (VSP) allows three-dimensional assessment of complex dentofacial deformities and has become integral to modern orthognathic surgery. However, evidence remains limited regarding how skeletal characteristics and malocclusion patterns translate into surgical movement selection. This study aimed to evaluate demographic features, skeletal malocclusion patterns, and clinical treatment strategies in patients undergoing VSP-guided orthognathic surgery. **Methods:** This retrospective study included 158 patients who underwent VSP-assisted orthognathic surgery between 2019 and 2025. Sagittal skeletal classification, vertical growth pattern, facial asymmetry, and maxillary crossbite were evaluated together with planned maxillary and mandibular movements. Surgical procedures were analyzed according to skeletal malocclusion classes (Class I, II, and III). Group comparisons were performed using chi-square and Kruskal–Wallis tests. Multivariable logistic regression analysis was conducted to assess factors associated with bimaxillary surgery (*p* < 0.05). **Results:** Skeletal Class I malocclusion was most prevalent (46.8%), followed by Class III (29.7%) and Class II (23.4%). Hyperdivergent growth patterns were predominantly observed in Class II patients, whereas normodivergent patterns were most common in Class III cases (*p* < 0.05). Mandibular advancement and setback generally followed expected class-based trends but were also observed across non-corresponding skeletal classes. Maxillary impaction and mandibular autorotation were frequently incorporated. Bimaxillary surgery was performed in 84.2% of cases. Logistic regression analysis showed no independent predictors of bimaxillary surgery (*p* > 0.05). **Conclusions:** VSP-assisted orthognathic surgery demonstrates that surgical planning cannot be reduced to sagittal skeletal classification alone. Treatment decisions are shaped by combined sagittal, vertical, transverse, and patient-specific factors, supporting a multidimensional and individualized planning approach.

## 1. Introduction

Dentofacial deformity has been defined as a condition primarily affecting the jaw and dental structures and impacting a variable proportion of the population. As a result of these deformities, numerous head and neck functions—including breathing, swallowing, speech, and mastication—may be adversely affected [1,2]. While such deformities may be limited to a single jaw, they can also involve multiple craniofacial structures. Although dentofacial deformities are relatively common in the general population, only a subset of affected individuals requires orthognathic surgery as definitive treatment. While some epidemiological studies have suggested that dentofacial discrepancies may affect a considerable proportion of the population, the exact prevalence of cases requiring surgical correction remains uncertain [3,4].

Orthognathic surgery is a generally safe and versatile set of procedures that addresses significant functional and esthetic discrepancies. During these operations, the maxilla and mandible are aligned in predetermined positions to achieve proper skeletal and dental positioning [5,6]. These procedures not only result in functional and cosmetic improvements but also provide psychological and social benefits to the patient through enhanced facial appearance [7,8].

Orthognathic surgery is typically indicated when dentofacial deformities cannot be adequately corrected with orthodontic treatment alone. It is most commonly considered for pronounced skeletal Class II or Class III discrepancies, vertical problems such as open bite or deep bite, facial asymmetry, and functional complaints that may compromise mastication, speech, or airway function. Importantly, appropriate patient selection remains fundamental to achieving predictable outcomes. Medical comorbidities requiring optimization, compromised periodontal health, and pre-existing temporomandibular joint-related symptoms may necessitate individualized risk assessment and careful perioperative planning. Accordingly, comprehensive evaluation and close interdisciplinary coordination between orthodontists and maxillofacial surgeons are essential for establishing realistic treatment goals and optimizing clinical outcomes [9,10,11].

In most cases, orthognathic surgery is integrated with pre- and postoperative orthodontic treatment. The success of surgical treatment depends on the accurate diagnosis of the dentofacial deformity and the formulation of a treatment plan that is feasible in the operating room. The effectiveness of the treatment plan, in turn, requires a thorough physical examination and a comprehensive data collection process [12,13].

Since the late 20th century, advancements in computer-aided design/manufacturing (CAD/CAM) systems and three-dimensional design technologies have enabled the rapid development and widespread adoption of virtual surgical planning (VSP) in orthognathic surgery [14]. In recent years, VSP has become an essential component of modern orthognathic surgery workflows. Several recent studies have demonstrated that VSP improves surgical accuracy and predictability by enabling detailed three-dimensional visualization of craniofacial structures and allowing precise transfer of planned movements to the operating room. In addition, digital workflows incorporating CAD/CAM technologies and patient-specific surgical guides have been reported to enhance planning efficiency and interdisciplinary communication between surgeons and orthodontists. These technological developments have further reinforced the role of VSP in contemporary orthognathic surgical decision-making [14,15,16,17].

VSP integrates digital dental models with three-dimensional skeletal imaging on a single platform, enabling simultaneous visualization of the dental arches and surrounding bony structures. This consolidated 3D environment improves diagnostic recognition and planning of complex movements—including yaw, cant, pitch, and bodily translations—and supports a more comprehensive assessment of craniofacial asymmetry that may be obscured in conventional two-dimensional workflows. During mandibular positioning, contemporary VSP workflows also emphasize verification of condylar seating within the glenoid fossa and can improve intraoperative control of centric relation. Because orthognathic surgical changes in the mandibular position and occlusal plane can alter TMJ loading and compressive stresses, optimizing preoperative records and correcting condylar seating/CR-related discrepancies may help reduce postoperative biomechanical stress on the TMJ [16,17,18]. As cases of condylar displacement and resorption have been reported in the literature following orthognathic surgery [19], the diagnostic contributions of VSP provide a significant advantage in improving the accuracy and reproducibility of orthognathic treatment planning [16].

Although several studies have investigated the prevalence and general characteristics of patients undergoing orthognathic surgery, most have primarily focused on sagittal skeletal discrepancies or overall treatment trends. For instance, previous studies have reported that skeletal Class III malocclusion is frequently observed among orthognathic surgery patients and have described general treatment patterns used in different populations. However, the distribution of additional clinical parameters and their relationship with the selection of specific orthognathic surgical movements have not been sufficiently explored [20,21]. In particular, how vertical growth patterns, facial asymmetry, and transverse discrepancies interact with sagittal malocclusion types during surgical planning remains unclear.

Virtual surgical planning (VSP) systems provide detailed quantitative outputs; however, the interpretation of isolated landmark displacement values may be misleading in complex orthognathic procedures. Combined translational and rotational movements, such as mandibular autorotation following maxillary impaction, may produce positional changes that do not directly correspond to the primary surgical movement performed. Therefore, evaluating surgical strategies within a multidimensional framework that integrates sagittal, vertical, and transverse relationships may provide more meaningful insight into clinical decision-making patterns. Accordingly, the aim of the present study was to investigate treatment-planning tendencies in VSP-guided orthognathic surgery by analyzing how skeletal malocclusion patterns and associated clinical characteristics influence the selection of surgical movements. The primary outcome variable was the selection of specific surgical movements (performed vs. not performed), reflecting clinical decision-making patterns in VSP-guided planning.

## 2. Materials and Methods

The study was approved by the Ethics Committee of the Faculty of Dentistry at Ankara University (Approval Date: 5 May 2025, Approval No: 9/22). This retrospective study reviewed the records of 158 patients who underwent VSP at the Department of Orthodontics and received orthognathic surgery at the Department of Oral and Maxillofacial Surgery, Ankara University, between 2019 and 2025.

During the preparation of this manuscript, the authors used ChatGPT (OpenAI, version 5.2, San Francisco, CA, USA) and Rubriq (https://rubriq.com/, Rubriq, Durham, NC, USA) for translation and language editing. The authors reviewed and edited the generated content and take full responsibility for the final content of this publication.

**Figure 1:**
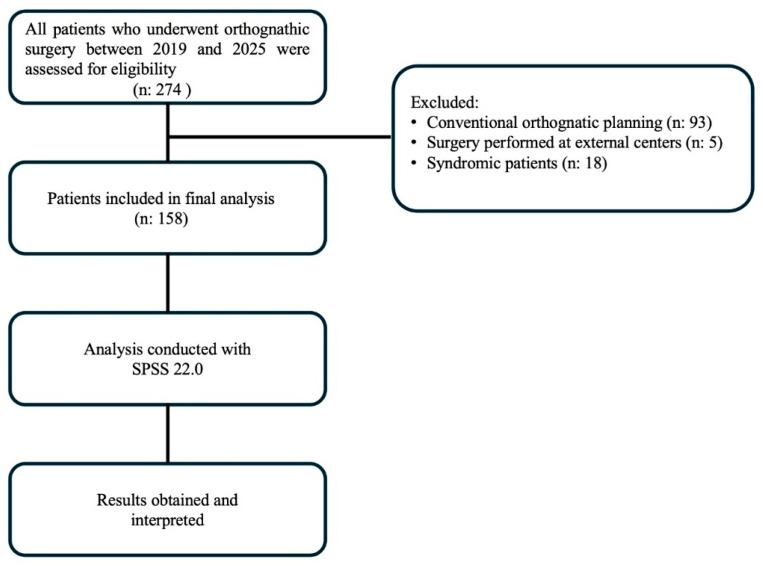
Flowchart of sample selection and evaluation process.

Patients who underwent conventional orthognathic planning, those whose surgeries were performed at other centers, and individuals with cleft lip and palate anomalies, previous orthognathic surgery, major facial trauma involving the jaws, syndromic craniofacial anomalies, or clinically significant temporomandibular joint pathology requiring active treatment were excluded from the study population. The steps related to sample selection and evaluation are presented in Figure 1.

The decision regarding pre-surgical orthodontics versus a surgery-first approach was made by the orthodontist based on the initial study models. The surgery-first approach was selected for patients with a stable occlusal relationship and no clinically significant crowding at the initial evaluation. Patients who did not fulfill these criteria underwent conventional pre-surgical orthodontic preparation.

The diagnoses related to dentofacial deformities included vertical deficiency or excess, transverse maxillary deficiency, sagittal discrepancies, and mandibular asymmetry. The surgical procedures performed were evaluated by classifying them according to skeletal sagittal malocclusion types. In addition, the surgical stage, orthodontic treatment method, and surgical sequencing protocols were also assessed.

The study included patients whose surgical planning was performed using the three-dimensional VSP software NEMOFAB (version 2020; NEMOTEC SL, Madrid, Spain). For virtual surgical planning, all required records, including CBCT data and STL files of the dental models, were obtained for each patient. Surgical planning was performed using NemoFab three-dimensional planning software, followed by the fabrication of surgical splints. Within the software environment, the CBCT data and STL dental models were registered to generate an integrated three-dimensional craniofacial model, and natural head posture was established. After registration, temporomandibular joint and airway measurements were performed based on the radiographic data. The models were then segmented, osteotomy lines were defined, and both two-dimensional and three-dimensional cephalometric analyses were carried out to finalize the surgical plan.

All surgical plans were generated within a comprehensive three-dimensional planning framework in which maxillary and mandibular movements were evaluated across sagittal, vertical, and transverse dimensions, together with rotational parameters (yaw, pitch, and roll). During the planning process, skeletal movements were digitally recorded as vectors for each patient. However, because combined translational and rotational movements may produce complex positional changes in individual landmarks, the analytical classification in the present study was based on the presence or absence of specific surgical movements rather than the magnitude of displacement. Accordingly, surgical movements were categorized on a per-patient basis as “movement performed” or “no movement.” Movements smaller than 0.5 mm were excluded from analysis as they were considered clinically insignificant. The resulting data were subsequently analyzed using comparative statistical methods according to skeletal malocclusion classifications (Class I, Class II, and Class III).

The evaluation of maxillary crossbite was based on the measurement of buccal overjet. A buccal overjet of ≤−1 mm was considered the diagnostic threshold for identifying maxillary crossbite.

Asymmetry was assessed based on a Menton deviation measured from cone-beam computed tomography (CBCT) images. A deviation of ≥4 mm from the midline plane was considered clinically significant facial asymmetry.

To evaluate the effects of vertical maxillary movements in isolation, impaction or inferior repositioning was considered present only if these movements were applied to the entire maxilla, involving both the anterior and posterior regions.

To determine the sagittal skeletal relationships of the patients, they were categorized into three groups based on the ANB angle—a cephalometric measurement formed between Point A, Nasion, and Point B—that assesses the relative sagittal position of the maxilla and mandible: Class I (0° ≤ ANB ≤ 4°), Class II (ANB > 4°), and Class III (ANB < 0°). For the evaluation of vertical skeletal relationships, the angle between the Gonion–Gnathion (GoGn) plane and the Sella–Nasion (SN) plane, which represents the cranial base, was used. Based on the GoGn-SN angle, patients were classified into three groups: normal growth pattern (28° ≤ GoGn-SN ≤ 36°), high-angle (GoGn-SN > 36°), and low-angle (GoGn-SN < 28°).

### Statistical Analysis

Statistical analyses were performed using IBM SPSS Statistics software (version 22.0; IBM Corp., Armonk, NY, USA). Descriptive statistics were presented as frequencies and percentages for categorical variables. Associations between skeletal malocclusion classes and the types of surgical procedures as well as morphological characteristics were evaluated using the Pearson chi-square test, where appropriate. In analyses with low expected cell frequencies, Fisher’s exact test was applied to obtain more reliable estimates of statistical significance. For continuous variables that did not follow a normal distribution, comparisons among three groups were performed using the Kruskal–Wallis H test. To evaluate independent preoperative factors associated with the selection of bimaxillary surgery, a multivariable binary logistic regression analysis was conducted. In this model, the surgical approach was defined as the dependent variable (0 = single-jaw surgery, 1 = bimaxillary surgery). The independent variables included skeletal malocclusion class (reference category: Class I), vertical growth pattern (reference category: normodivergent), presence of facial asymmetry, and presence of maxillary crossbite. All variables were entered into the model using the Enter method. Multicollinearity among the independent variables was assessed using the variance inflation factor (VIF). Model fit was evaluated using the Hosmer–Lemeshow goodness-of-fit test. Regression results were reported as odds ratios (ORs) with 95% confidence intervals and corresponding *p*-values. A two-sided *p*-value of <0.05 was considered statistically significant for all analyses.

A post hoc power analysis was performed using G*Power software (version 3.1, Heinrich Heine University, Düsseldorf, Germany) to evaluate the adequacy of the sample size. Based on a medium effect size (effect size = 0.3), an alpha level of 0.05, and a statistical power of 0.80, the minimum required sample size was calculated as 108 participants. As the present study included 158 patients, the sample size was considered sufficient for the planned analyses.

Measurements of facial asymmetry, crossbite, and growth pattern were performed by the same investigator (M.D.) using the preoperative VSP records. To evaluate measurement reliability, 30 randomly selected cases were re-measured by the same investigator two weeks after the initial assessment. Intra-observer reliability for these measurements was assessed using the intraclass correlation coefficient (ICC).

## 3. Results

The intra-observer reliability analysis demonstrated excellent agreement, with ICC values ranging from 0.89 to 0.95. A total of 158 patients underwent VSP-assisted orthognathic surgery during the study period. The mean age was 27.8 years (Table 1), and females represented the majority of the cohort (Table 2).

Skeletal Class I malocclusion was the most common pattern, followed by Class III and Class II. When evaluated according to sagittal and vertical skeletal relationships, the hyperdivergent growth pattern was predominant in the Class II group (73.0%), whereas the normodivergent pattern was more prominent in the Class III group (70.2%) (*p* = 0.0001). No statistically significant differences were found in the distribution of sagittal and vertical skeletal relationships by sex (*p* > 0.05).

A statistically significant correlation was found between Angle dental classification and skeletal malocclusion (χ^2^ = 52.5, *p* = 0.0001). Most skeletal Class III individuals corresponded to Angle Class III, whereas skeletal Class II patients were predominantly classified as Angle Class II.

Facial asymmetry was observed in 66.5% of the patients, but no statistically significant association was found with the skeletal malocclusion type (*p* = 0.179).

Bilateral and circular crossbites were more frequently observed in Class III patients; however, the association was not statistically significant (*p* = 0.079) (Table 3).

A statistically significant association was observed between mandibular advancement and skeletal malocclusion classes (*p* = 0.0001). Mandibular advancement was most frequently performed in Class II cases (70.3%), although it was also observed in 14.9% of Class III patients. A significant association was also found between mandibular setback and skeletal malocclusion classes (*p* = 0.0001), with the highest prevalence in Class III cases (83.0%), while 32.4% of Class II patients also underwent mandibular setback. Mandibular autorotation was more frequent in skeletal Class I patients (70.3%) and was included in the surgical planning of 62% of the overall cohort; however, this association did not reach statistical significance (*p* = 0.098).

No statistically significant association was found between maxillary impaction and skeletal malocclusion classes (*p* = 0.094), although it was relatively more common in Class II patients (Table 4).

Bimaxillary surgery was the most commonly performed procedure, followed by Le Fort I osteotomy. Bimaxillary surgery was most frequently performed in patients with skeletal Class II malocclusion (89.2%), followed by those with skeletal Class I and Class III malocclusions. Among patients with skeletal Class III malocclusion, the rate of bimaxillary surgery was lower than in Class I and II groups; however, the difference was not statistically significant.

The orthodontics-first protocol was the most commonly applied treatment approach. Fixed appliances were the predominant orthodontic modality, whereas clear aligners were used in a smaller proportion of cases (Table 5).

Multivariable logistic regression analysis was performed to evaluate factors associated with bimaxillary surgery. Skeletal malocclusion class, vertical growth pattern, presence of facial asymmetry, and presence of maxillary crossbite were included in the model. None of the evaluated variables were identified as independent predictors of bimaxillary surgery (*p* > 0.05 for all variables). A trend toward lower odds of bimaxillary surgery was observed in skeletal Class III patients, although the findings did not reach statistical significance (Table 6).

## 4. Discussion

This study provides a comprehensive overview of skeletal characteristics and clinical treatment patterns in patients undergoing orthognathic surgery planned with VSP. The findings highlight the multidimensional nature of dentofacial deformities and the frequent need for combined surgical movements.

The sex distribution of patients undergoing orthognathic surgery may vary depending on the population studied. In the present study, the majority of cases consisted of young female patients, with a mean age of 27.7 years. These findings are consistent with those reported by Olkun et al. in a Turkish population and by Sato et al. in Brazil [22,23]. Nonetheless, some studies have reported a comparable or even higher proportion of male patients compared to females [24,25].

Although numerous studies in the literature have reported skeletal Class III malocclusion as the most prevalent pattern among patients undergoing orthognathic surgery [25,26,27], the most frequently operated individuals in the present study exhibited skeletal Class I malocclusion. As in many previous studies, skeletal classification in this study was based on the ANB angle; however, it should be acknowledged that the ANB angle may be influenced by factors such as the position of the nasion point and lower facial height.

In patients with a skeletal Class I sagittal relationship, deformities in other planes—such as maxillary or mandibular rotations, facial asymmetry, vertical excess or deficiency, and transverse constriction—often constitute the primary indication for surgical intervention. In addition, population characteristics, referral patterns (particularly esthetic concerns), and variations in clinical decision-making processes may have contributed to this distribution. Accordingly, the most frequently operated patients in this study presented with skeletal Class I malocclusion and Angle Class III malocclusion.

Although skeletal and dental malocclusions are often evaluated together, it has been shown that these two components do not always coincide [28]. In the present study, a statistically significant association was found between Angle classification and skeletal malocclusion classes. While 93.6% of patients with skeletal Class III malocclusion presented with Angle Class III malocclusion, only 64.9% of patients with skeletal Class II malocclusion exhibited Angle Class II malocclusion. In contrast, the prevalence of Angle Class I malocclusion in the skeletal Class I group was notably low (2.7%), with the majority of these patients presenting with Angle Class III malocclusion (68.9%). These findings underscore that dental and skeletal classifications should not be used interchangeably and highlight the importance of a multidimensional assessment approach in surgical planning.

Class II malocclusions are often associated with an increased mandibular plane angle and increased vertical facial height, indicating a hyperdivergent growth pattern [29]. In the majority of Class III cases, the mandibular plane is positioned more horizontally, and a normodivergent or hypodivergent growth pattern tends to predominate. However, a study conducted by Olkun et al. in Turkey reported a higher prevalence of skeletal Class III malocclusion associated with a high-angle vertical growth pattern [23]. In the present study, the hyperdivergent growth pattern was predominant in the Class II group, whereas the normodivergent growth pattern was more prominent in the Class III group. Vertical growth patterns differed significantly among the skeletal malocclusion classes. These differences suggest that the vertical growth pattern is not merely an accompanying characteristic of sagittal skeletal classification but may also play a determining role in patient selection and in defining surgical indications, as also emphasized in the orthognathic literature [30].

Facial asymmetry is considered a significant indication for orthognathic surgery [31]. In the study conducted by Severt and Proffit, facial asymmetry was identified in 34% of the patients, and it was noted as one of the primary reasons for seeking surgical treatment [32]. Similarly, Sato et al. emphasized the high prevalence of facial asymmetry in individuals undergoing orthognathic surgery in conjunction with orthodontic treatment, reporting the presence of asymmetry in 46.7% of their samples [22]. In the present study, facial asymmetry was observed in a considerable proportion of patients undergoing orthognathic surgery, supporting previous reports that asymmetry represents an important indication for surgical correction.

In this study, while the high frequency of maxillary advancement in skeletal Class III cases is an expected finding, the very high rate of maxillary advancement planned in the Class II group (91.9%) is noteworthy. This pattern may be explained by multiple clinical parameters acting concurrently in surgical planning, including the possibility of concomitant maxillary retrusion in a subset of Class II patients, soft-tissue esthetic objectives, stability limits of mandibular advancement, the need for occlusal plane and/or rotational corrections, and the frequent use of maxillary impaction in association with a hyperdivergent growth pattern [33,34].

Mandibular advancement is the standard surgical approach for Class II malocclusion, whereas mandibular setback is most frequently indicated in Class III cases [22,24,35]. In the present study, mandibular movements generally followed these expected patterns. However, the presence of mandibular advancement in some Class III patients suggests that sagittal skeletal classification alone does not fully determine the surgical vector. Instead, orthognathic surgical planning appears to be influenced by multiple three-dimensional parameters, including asymmetry, rotational requirements, and occlusal plane objectives [36,37,38].

Similarly, although mandibular setback was most frequently associated with skeletal Class III malocclusion, its occurrence in some Class II cases indicates that clinical decision-making cannot be reduced solely to ANB-based skeletal classification [18]. These findings suggest that orthognathic surgical planning is influenced by multiple patient-specific three-dimensional factors, including asymmetry, vertical pattern, rotational requirements, and soft-tissue considerations [39]. A conceptual treatment-planning model derived from these relationships is presented in Supplementary Figure S1.

In skeletal Class III cases, bimaxillary surgery was frequently preferred, suggesting that sagittal discrepancies in this group were commonly addressed through a combined surgical approach. Previous studies have reported that bimaxillary surgery may provide more favorable functional and esthetic outcomes than isolated mandibular setback in selected Class III patients [40,41]. In contrast, the predominance of mandibular advancement in the Class II group suggests that mandibular retrognathia may represent the main skeletal component in many of these cases.

However, despite the descriptive between-group differences in the preference for bimaxillary surgery, the multivariable logistic regression model constructed to predict the likelihood of undergoing bimaxillary surgery showed that none of the included variables—skeletal malocclusion class (Class II and Class III), vertical growth pattern (hypodivergent/hyperdivergent), presence of facial asymmetry, or presence of maxillary crossbite—were statistically significant independent predictors (all *p* > 0.05). Nevertheless, a trend toward lower odds of bimaxillary surgery was observed in skeletal Class III cases. Although the presence of facial asymmetry showed a trend toward increased odds of bimaxillary surgery, this association did not reach statistical significance. Likewise, a hyperdivergent growth pattern and the presence of maxillary crossbite were not significantly associated with bimaxillary surgery (Table 6). These findings suggest that the decision between single- and bimaxillary surgery is influenced by multiple factors beyond the variables included in the model, such as deformity severity, occlusal plane objectives, and surgeon preference. The wide confidence intervals further indicate that the model may have had limited power to detect potential associations.

Another notable observation in the present cohort is the high prevalence of bimaxillary surgery across all sagittal malocclusion groups. Bimaxillary procedures accounted for the majority of cases (84.2%) and were frequently selected not only in Class III patients but also in Class I and Class II groups. Consistent with this multidimensional perspective, bimaxillary surgery was the most frequently performed procedure across all sagittal groups in our cohort. This observation suggests that orthognathic surgical planning cannot be explained solely by sagittal skeletal classification. Instead, vertical skeletal patterns, transverse discrepancies, and facial asymmetry often necessitate combined maxillary and mandibular interventions to achieve optimal functional and esthetic outcomes. These findings highlight the multidimensional nature of orthognathic treatment planning and indicate that relying exclusively on sagittal classification may oversimplify the complexity of clinical decision-making. One possible explanation for the relatively high frequency of bimaxillary procedures in our cohort may be the increasing emphasis on preserving or improving upper airway dimensions during orthognathic treatment planning. Mandibular setback procedures performed in isolation have been associated with potential reductions in posterior airway space, whereas combined maxillary and mandibular surgical approaches may allow more balanced skeletal repositioning while minimizing adverse airway effects [42]. For this reason, contemporary surgical planning in many centers increasingly favors bimaxillary approaches even in cases where a single-jaw procedure might appear sufficient based solely on sagittal classification.

Mandibular autorotation may be preferred following maxillary impaction, as it can reduce lower facial height, improve the esthetic profile, or, in some cases, eliminate the need for bimaxillary surgery. However, the clinical implications and role of this phenomenon in surgical planning remain controversial in the literature and among clinicians due to the difficulty in predicting the direction and magnitude of autorotation, the potential risk of increased load on the TMJ, and the uncertainty regarding its long-term stability [42,43,44,45]. In the present study, mandibular autorotation was performed in 62% of the cases, almost all of which were observed in conjunction with maxillary impaction.

In the evaluation of maxillary crossbite distribution, crossbite was infrequently observed among individuals with Class II skeletal patterns, whereas bilateral and circular crossbites were predominantly identified in Class III cases. This pattern may be explained by the relative influence of sagittal skeletal discrepancies on transverse occlusal relationships. In particular, the anterior positioning of the mandible in Class III individuals may exaggerate underlying transverse disharmonies, thereby contributing to the clinical manifestation of crossbite. These findings support the notion that sagittal jaw discrepancies influence transverse occlusal relationships in a relative—rather than absolute—manner [46,47].

Surgical sequencing in bimaxillary orthognathic surgery remains a debated topic in the literature, and no universal consensus exists regarding which jaw should be operated on first to achieve optimal outcomes [48,49,50]. The maxilla-first approach is generally preferred because of its surgical advantages, particularly in cases requiring maxillary impaction, whereas the mandible-first sequence may be used in selected situations such as planned anticlockwise rotation or segmental Le Fort I osteotomy [51,52]. Consistent with these considerations, the maxilla-first approach was the predominant surgical protocol in the present study.

Orthognathic surgery has traditionally followed a sequential treatment protocol consisting of preoperative orthodontics, surgery, and postoperative orthodontic treatment [53,54]. However, this conventional approach may involve prolonged treatment duration and temporary deterioration in facial esthetics during the presurgical orthodontic phase [42]. With advances in technology and improved understanding of skeletal stability, alternative strategies such as surgery-first and surgery-only protocols, as well as the use of clear aligners, have emerged. These approaches may offer advantages, including reduced treatment time and improved patient satisfaction in appropriately selected cases [53].

These findings may have important clinical implications for orthognathic treatment planning. The results of the present study suggest that surgical decision-making should not rely solely on skeletal malocclusion classification but should also incorporate three-dimensional craniofacial characteristics, facial asymmetry, and occlusal objectives. Considering these factors together may support more individualized treatment planning and potentially improve functional and esthetic outcomes.

One of the major strengths of this study is the relatively large patient sample (*n* = 158) in which VSP was utilized, with case analyses performed in a standardized manner using three-dimensional planning software. In addition, skeletal malocclusions were evaluated not only in the sagittal plane but also in conjunction with vertical and transverse parameters. The incorporation of asymmetry, growth patterns, and surgical movements into multidimensional statistical analyses enabled the study to generate clinically relevant data that may support surgical decision-making. Although VSP systems allow the quantification of skeletal displacement in millimeters or degrees, the positional change in individual landmarks may not always accurately reflect the actual surgical movement due to interactions between translational and rotational components. For example, mandibular setback combined with maxillary impaction may induce mandibular autorotation, resulting in apparent anterior positional changes in landmarks such as the B-point despite posterior mandibular translation. For this reason, the present study focused on treatment-selection patterns rather than the magnitude of skeletal displacement. Nevertheless, certain limitations should be acknowledged. The retrospective design may introduce potential sources of bias, including patient referral patterns, esthetic expectations, or clinician preferences that could not be controlled during case selection. Additionally, the use of data from a single center may limit the generalizability of the findings, and the analysis of surgical movements in binary categories (performed/not performed) does not account for movement magnitude. Future prospective, multicenter studies using standardized coordinate systems are therefore warranted to further investigate quantitative displacement values in relation to orthognathic surgical planning.

## 5. Conclusions

In this retrospective study, demographic characteristics, types of dentofacial deformities, and planned surgical movements were comprehensively evaluated in patients undergoing orthognathic surgery guided by VSP. The findings demonstrated that the majority of patients were young adult females and that the most frequently observed pattern was a combination of skeletal Class I and dental Angle Class III malocclusion.

The observation that mandibular advancement was also performed in Class III cases, while mandibular setback was preferred in a subset of Class II patients, indicates that the selection of surgical movements cannot be reduced to sagittal skeletal classification alone. Together with the widespread use of maxillary impaction and mandibular autorotation, and the frequent coexistence of sagittal deformities with vertical and transverse components, these findings underscore the necessity of a multidimensional approach to treatment planning.

The three-dimensional analytical capabilities provided by VSP facilitated a more accurate assessment of the complex nature of jaw relationships and supported the development of patient-specific surgical strategies. In this context, VSP-assisted orthognathic surgery offers clinically meaningful advantages for achieving anatomical harmony, optimizing functional outcomes, and enhancing esthetic satisfaction.

## Figures and Tables

**Table 1:** Descriptive statistics and non-parametric analysis of age distribution across skeletal malocclusion classes.

		*n*	Mean	Median	Minimum	Maximum	ss	*p* *
Skeletal malocclusion	Class 1	74	28.0	26.3	19.7	44.7	5.2	0.316
	Class 2	37	28.1	27.3	20.3	37.3	5.1	
	Class 3	47	27.1	25.5	17.3	55.9	7.2	
	Total	158	27.8	26.3	17.3	55.9	5.8	

*n*: number of participants; * Kruskal–Wallis H test significance value.

**Table 2:** Sex distribution by percentage in the overall patient cohort.

Sex	*n*	%
Female	93	58.9
Male	65	41.1
Total	158	100.0

*n*: number of participants.

**Table 3:** Skeletal malocclusion-based distribution of asymmetry, vertical pattern, angle classification, and maxillary crossbite.

		Class 1		Class 2		Class 3		Total		*p* *
		*n*	%	*n*	%	*n*	%	*n*	%	
Asymmetry	Absent	26	35.1	8	21.6	19	40.4	53	33.5	0.179
	Present	48	64.9	29	78.4	28	59.6	105	66.5	
	Total	74	100.0	37	100.0	47	100.0	158	100.0	
Vertical Growth Pattern	Normodivergent	43	58.1	10	27.0	33	70.2	86	54.4	0.0001
	Hypodivergent	5	6.8	0	0.0	3	6.4	8	5.1	
	Hyperdivergent	26	35.1	27	73.0	11	23.4	64	40.5	
	Total	74	100.0	37	100.0	47	100.0	158	100.0	
Angle Classification	Angle 1	2	2.7	2	5.4	0	0.0	4	2.5	0.0001
	Angle 2	14	18.9	24	64.9	2	4.3	40	25.3	
	Angle 3	51	68.9	8	21.6	44	93.6	103	65.2	
	Angle 4	7	9.5	3	8.1	1	2.1	11	7.0	
	Total	74	100.0	37	100.0	47	100.0	158	100.0	
Maxillary Crossbite	Absent	28	37.8	22	59.5	12	25.5	62	39.2	0.079
	Right	10	13.5	5	13.5	9	19.1	24	15.2	
	Left	10	13.5	5	13.5	5	10.6	20	12.7	
	Bilateral	21	28.4	5	13.5	17	36.2	43	27.2	
	Circular	5	6.8	0	0.0	4	8.5	9	5.7	
	Total	74	100.0	37	100.0	47	100.0	158	100.0	

*n*: number of participants; * Chi-square significance value.

**Table 4:** Maxillary and mandibular surgical movements by skeletal malocclusion type.

		Class 1		Class 2		Class 3		Total		*p* *
		*n*	%	*n*	%	*n*	%	*n*	%	
Maxillary Impaction	Absent	15	21.7	6	17.6	16	37.2	37	25.3	0.094
	Present	54	78.3	28	82.4	27	62.8	109	74.7	
Maxillary Downgrafting	Absent	62	89.9	27	79.4	36	81.8	125	85.0	0.292
	Present	7	10.1	7	20.6	8	18.2	22	15.0	
Maxillary Advancement	Absent	7	9.5	2	5.4	7	14.9	16	10.1	0.281
	Present	67	90.5	34	91.9	40	85.1	141	89.2	
	Setback	0	0.0	1	2.7	0	0.0	1	0.6	
Maxillary Rotation	Absent	35	47.3	15	40.5	19	40.4	69	43.7	0.689
	Present	39	52.7	22	59.5	28	59.6	89	56.3	
Maxillary Translation	Absent	33	44.6	13	35.1	21	44.7	67	42.4	0.593
	Present	41	55.4	24	64.9	26	55.3	91	57.6	
Mandibular Advancement	Absent	48	64.9	11	29.7	40	85.1	99	62.7	0.0001
	Present	26	35.1	26	70.3	7	14.9	59	37.3	
Mandibular Setback	Absent	21	28.4	25	67.6	8	17.0	54	34.2	0.0001
	Present	53	71.6	12	32.4	39	83.0	104	65.8	
Mandibular Rotation	Absent	28	37.8	15	40.5	20	42.6	63	39.9	0.871
	Present	46	62.2	22	59.5	27	57.4	95	60.1	
Mandibular Translation	Absent	22	29.7	6	16.2	15	31.9	43	27.2	0.221
	Present	52	70.3	31	83.8	32	68.1	115	72.8	
Mandibular Autorotation	Absent	22	29.7	15	40.5	23	48.9	60	38.0	0.098
	Present	52	70.3	22	59.5	24	51.1	98	62.0	

*n*: number of participants; * Chi-square significance value.

**Table 5:** Distribution of parameters related to the orthognathic treatment process by skeletal classification.

		Class 1		Class 2		Class 3		Total		*p* *
		*n*	%	*n*	%	*n*	%	*n*	%	
Surgery Stage	Surgery First	4	5.4	3	8.1	3	6.4	10	6.3	0.247
	Orthodontics First	68	91.9	31	83.8	38	80.9	137	86.7	
	Only Surgery	2	2.7	3	8.1	6	12.8	11	7.0	
Orthodontic Treatment Method	Clear Aligner	9	12.2	7	18.9	7	14.9	23	14.6	0.191
	Fixed Appliances	63	85.1	27	73.0	34	72.3	124	78.5	
	None	2	2.7	3	8.1	6	12.8	11	7.0	
Surgery Sequence	Maxilla First	63	85.1	30	81.1	39	83.0	132	83.5	0.856
	Mandible First	11	14.9	7	18.9	8	17.0	26	16.5	
Surgical Procedure	Bimaxillary	65	87.8	33	89.2	35	74.5	133	84.2	0.184
	Only Maxilla	5	6.8	2	5.4	6	12.8	13	8.2	
	Only Mandible	4	5.4	1	2.7	6	12.8	11	7.0	
	Genioplasty	0	0.0	1	2.7	0	0.0	1	0.6	

*n*: number of participants; * Chi-square significance value.

**Table 6:** Multivariable logistic regression analysis of factors associated with bimaxillary surgery.

Variable	B	S.E.	*p*-Value	Odds Ratio (OR)	95% CI
Skeletal malocclusion					
Class II	−0.043	0.692	0.950	0.958	0.247–3.714
Class III	−0.883	0.498	0.076	0.413	0.156–1.098
Vertical growth pattern					
Hypodivergent	−0.407	0.906	0.653	0.666	0.113–3.929
Hyperdivergent	0.153	0.519	0.769	1.165	0.421–3.224
Facial asymmetry					
Present	0.635	0.455	0.163	1.886	0.774–4.598
Maxillary crossbite					
Present	0.064	0.484	0.895	1.066	0.413–2.755

B: regression coefficient; S.E.: standard error; OR: odds ratio; CI: confidence interval.

## Data Availability

The data presented in this study are available on request from the corresponding author. The data are not publicly available due to privacy restrictions.

## Supplementary Materials

The following supporting information can be downloaded at https://www.mdpi.com/article/10.3390/healthcare14060809/s1. Figure S1: The model illustrates how sagittal classification interacts with vertical skeletal patterns, transverse discrepancies, and facial asymmetry during VSP-guided orthognathic planning, ultimately influencing the selection of surgical strategies.

## Abbreviations

The following abbreviations are used in this manuscript:

3D	Three-dimensional
ANOVA	Analysis of variance
CAD/CAM	Computer-aided design/manufacturing
CBCT	Cone-beam computed tomography
EC	Ethics Committee
GoGn	Gonion-Gnathion
LF 1	Le Fort 1
SD	Standard deviation
SN	Sella–Nasion
TMJ	Temporomandibular joint
VSP	Virtual surgical planning
