# Supplementary material for: Skeletal Characteristics and Clinical Treatment Patterns in Orthognathic Surgery: A Virtual Surgical Planning-Based Study
Source: Healthcare (Basel). 2026 Mar 22;14(6):809. doi: 10.3390/healthcare14060809 (PMC13026696; doi:10.3390/healthcare14060809)
Supplement: Supplementary file 1 [file healthcare-14-00809-s001.zip › healthcare-4187647-supplementary.pdf]

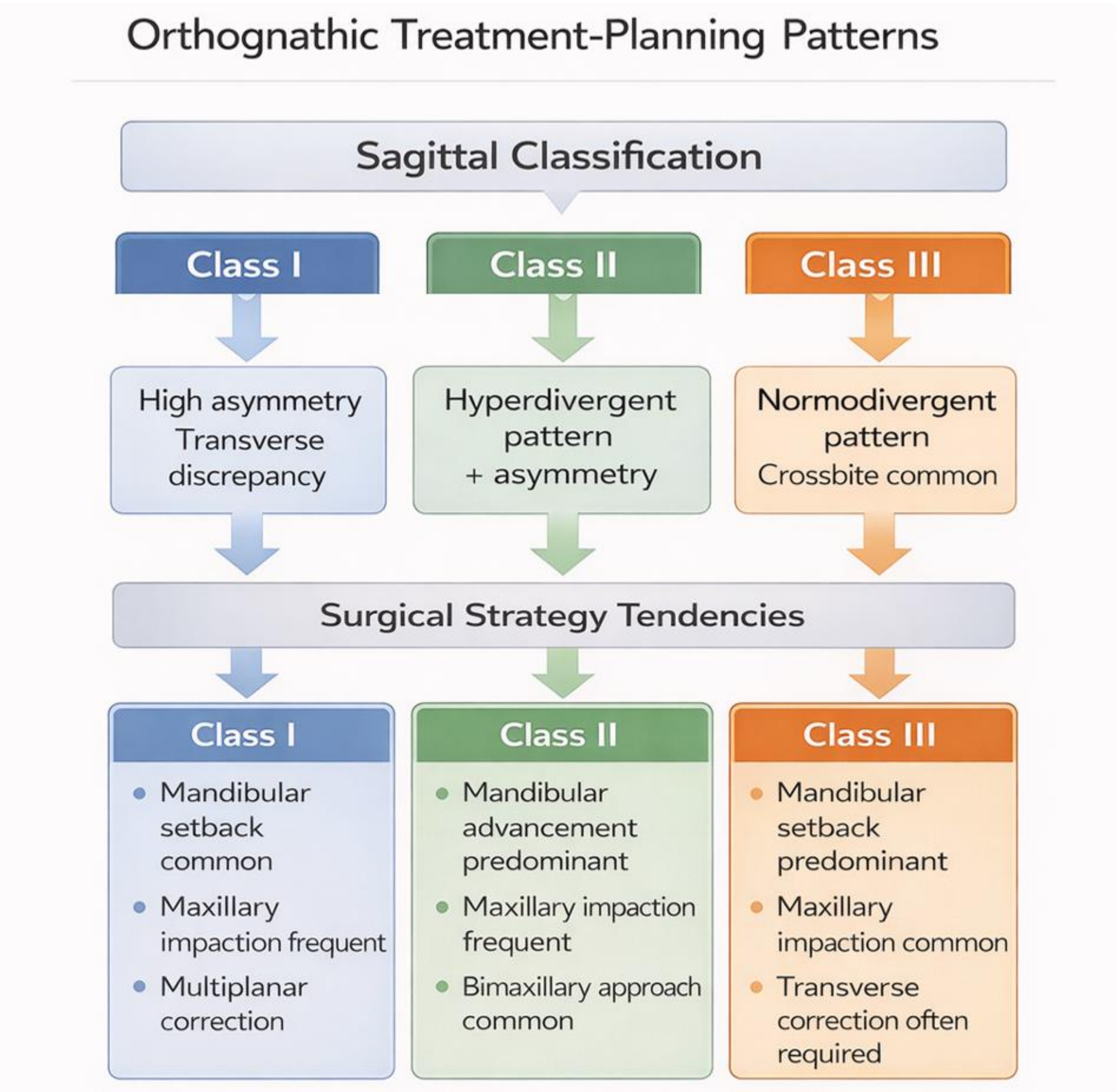

Supplementary Figure S1. The model illustrates how sagittal classification interacts with vertical skeletal patterns, transverse discrepancies, and facial asymmetry during VSP-guided orthognathic planning, ultimately influencing the selection of surgical strategies.
